# Intestinal dysbiosis featuring abundance of Streptococcus associates with Henoch-Schönlein purpura nephritis (IgA vasculitis with nephritis) in adult

**DOI:** 10.1186/s12882-021-02638-x

**Published:** 2022-01-03

**Authors:** Jiaxing Tan, Zhengxia Zhong, Yi Tang, Wei Qin

**Affiliations:** 1 West China Hospital, Sichuan University, Chengdu, Sichuan China; 2 West China School of Medicine, Sichuan University, Chengdu, Sichuan China; 3 Affiliated Hospital of Zunyi Medical University, Guizhou, China

**Keywords:** Henoch-Schönlein purpura nephritis (HSPN), Gut microbiota, Streptococcus

## Abstract

**Background:**

The pathogenesis of Henoch-Schönlein purpura nephritis (HSPN) is closely associated with mucosal infection. But whether intestinal microbiota dysbiosis plays a role in it is not clear.

**Methods:**

A total of 52 participants including 26 HSPN patients and 26 healthy controls were included. By using 16S ribosomal RNA gene sequencing, the intestinal microbiota composition between HSPN and healthy controls was compared. The diagnostic potency was evaluated by Receiver operating characteristic (ROC) with area under curves (AUC). Meanwhile, correlation analysis was also performed.

**Results:**

The lower community richness and diversity of fecal microbiota was displayed in HSPN patients and the structure of gut microbiota was remarkedly different. A genus-level comparison indicated a significant increase in the proportions of g-Bacteroides, g-Escherichia–Shigella and g-Streptococcus, and a marked reduction of g-Prevotella_9 in HSPN patients, suggesting that the overrepresentation of potential pathogens and reduction of profitable strains were the main feature of the dysbiosis. The differential taxonomic abundance might make sense for distinguishing HSPN from healthy controls, with AUC of 0.86. The relative abundance of the differential bacteria was also concerned with clinical indices. Among them, Streptococcus spp. was positively associated with the severity of HSPN (*P* < 0.050). It was found that HSPN patients with higher level of Streptococcus spp. were more likely to suffering from hematuria and hypoalbuminemia (*P* < 0.050).

**Conclusions:**

The dysbiosis of gut microbiota was obvious in HSPN patients, and the intestinal mucosal streptococcal infection was distinctive, which was closely related to its severity.

**Supplementary Information:**

The online version contains supplementary material available at 10.1186/s12882-021-02638-x.

## Introduction

Henoch-Schönlein purpura (HSP) or immunoglobulin A vasculitis (IgAV), characterized by cutaneous purpura, gastrointestinal disturbances, non-deforming arthritis and/or nephritis, is a frequent systemic small-vessel vasculitis affecting both children and adults [1, 2]. It has been thought that HSP was a self-limiting auto-immune disorder, with a largely good prognosis [3]. But renal involvement, also named Henoch-Schönlein purpura nephritis (HSPN), can cause severe outcomes, which is the major cause of mortality in patients with HSP [4]. The pathophysiological process of HSPN is unknown, but some scholars have proposed that genetic factors, environment and immune abnormalities have led to the occurrence and development of HSPN [5]. Allergies caused by food, infections, drugs and/or other allergens may be also associated with HSPN [6].

As the biggest immune organ in human body, the intestinal tract contains trillions of bacteria and immunocytes [7]. Emerging studies have proved that the gut-kidney axis may play an important role in the pathogenesis of many kidney diseases and the role that gut microbe is crucial for pathogenesis of kidney diseases has been increasingly researched since intestinal microbiota makes great contribution to shaping and modulating immune system responses [8–10]. It has been reported that dysbiosis of the gut microbial composition is correlated with various immune-mediated diseases, such as allergic asthma, inflammatory bowel disease, diabetic nephropathy, and chronic kidney diseases (CKD) [11–15].

Previous studies have reported that gut microbiota dysbiosis occurs in pediatric patients with HSP and adult patients with IgA nephropathy that shares the similar pathophysiological mechanism with HSPN [5, 16, 17]. However, no studies have been found to discuss the gut microbiota of adult patients with HSPN. Whether the intestinal bacteria have interaction with clinical manifestations of HSPN remains unknown. Therefore, we analyzed the gut microbes from excrement by sequencing the 16S ribosomal RNA (rRNA) gene, to explore the differences in the composition of gut microbiota in HSPN patients and healthy controls.

## Materials and methods

### Subjects

A total of 26 patients with a clinical and pathological diagnosis of HSPN in West China Hospital, Sichuan University, were recruited between June 2018 and November 2019. The criteria of diagnosis were mainly based on the European League Against Rheumatism (EULAR), Paediatric Rheumatology International Trials Organization (PRINTO) and Paediatric Rheumatology European Society (PRES) (EULAR/PRINTO/PRES) database and methodology [2, 18, 19]. Meanwhile, 26 healthy volunteers who had no other diseases estimated by researchers were enrolled as controls. All subjects should not be suffering from malnutrition, digestive tract diseases, autoimmune disorders, infectious diseases, abnormal liver function, malignancies and metabolic diseases. Notably, all individuals should receive no antibiotic treatment within 4 weeks and have no diseases or symptoms of digestive tract. This study was approved by the Biomedical Ethics Committee of West China Hospital of Sichuan University and conducted according to the Helsinki Declaration. Written informed consent from all participants were obtained.

The specimens were collected at the time of diagnosis when immunosuppressive therapy has not been performed. Each individual received an adequate dose of RAS inhibitor. All subjects received a unified diet 3 days before sampling.

Demographic and clinical data including sex, age, blood pressure, 24-h proteinuria, urinary red blood cells, hemoglobin, serum albumin, and creatinine were recorded. The Chronic Kidney Disease Epidemiology Collaboration (CKD-EPI) equation were used to calculate the estimated glomerular filtration rate (eGFR) [20]. There is currently no well-known pathological classification method for adult HSPN since International Study Group of Kidney Disease in Children (ISKDC) classification has great limitations on its usage in adult patients. Some scholars have suggested that Oxford Classification might be recommended for patients with HSPN, regardless of children and adults. Hence, Oxford Classification including mesangial proliferation (M), endocapillary proliferation (E), segmental glomerulosclerosis (S), tubular atrophy or interstitial fibrosis (T), and crescents (C) was used in this study. Enzyme linked immunosorbent assay (ELISA) kits (Shanghai Enzyme-linked Biotechnology Co., Ltd., China) were used to detect the levels of serum IgA and galactose-deficient IgA1 (Gd-IgA1).

### Sample size calculating

The G-Power software V3.1.9.7, as a flexible statistical power analysis program, was used to calculate the sample size [21]. The probability of committing a Type I error (alpha error probability) was 0.05 and the power (1 – beta error probability) was 0.80. The effect size, based on preliminary experimental results, was 0.80. The calculated sample size was 52, including 26 samples of HSPN and 26 samples of healthy controls. Therefore, the sample sizes of our study were adequate and reasonable.

### DNA extraction

A fresh fecal sample of each participant for genomic deoxyribonucleic acid (DNA) extraction was collected into an aseptic container and instantly stored at − 80 °C. The E.Z.N.A.® soil DNA Kit bought in Omega Bio-tek, Norcross, GA, U.S. was used to extract microbial DNA from the fecal samples, whose purity was verified by the NanoDrop 2000 UV-vis spectrophotometer (Thermo Scientific, Wilmington, USA). The size and the integrity of the final bacterial DNA were evaluated by the agarose gel electrophoresis.

### PCR amplification

The primer pair 338F (5′-ACTCCTACGGGAGGCAGCAG-3′) and 806R (5′-GGACTACHVGGGTWTCTAAT-3′) were applied to amplify the 16S ribosomal ribonucleic acid (16S rRNA) V3–4 region by thermocycler polymerase chain reaction (PCR) system (GeneAmp 9700, ABI, USA). The sequential processes consisted of denaturation (95 °C, 3 min), PCR cycles (27 cycles, 95 °C, 30 s), annealing (55 °C, 30 s), elongation (72 °C, 75 s) and extension (72 °C, 10 min). Then the PCR products extracted from agarose gel were further purified and quantified using the AxyPrep DNA Gel Extraction Kit (Axygen Biosciences, Union City, CA, USA) and QuantiFluor™ -ST (Promega, USA), respectively [11]. The amplified products that was pooled equimolarly, were sequenced on the Illumina MiSeq platform (Illumina, San Diego, CA, USA).

### Processing of sequencing data

Then, Trimmomatic was used to quality-filter the raw reads which was recorded by fastq files, and the sequencing read were merged by Flash. Flash was performed to merge the sequencing reads. By using UPARSE (version 7.1; http://drivve5.com/uparse), the sequencing reads with a 97% similarity cutoff were clustered into the same operational taxonomic units (OTUs), while the chimeric sequences were removed by UCHIME [22]. The ribosomal database project (RDP) classifier algorithm (http://rdp.cme.msu.edu/) against the Silva (SSU123) 16S rRNA database with a70% confidence threshold was used to analyzed the taxonomy of each representative sequence of OTUs.

### Statistical analysis

Demographic information and laboratory data at baseline were presented as means ± standard deviation, medians with interquartile range, or numbers with percentages. The Student’s t-test, Wilcoxon rank-sum test, ANOVA, nonparametric Mann–Whitney U test, chi-square test, or Fisher’s exact test calculated by SPSS version 26.0 (IBM SPSS, Chicago, IL, USA) were selectively performed to compare the differences between the groups, according to the data type and distribution. Two-tailed *P* value < 0.05 was considered statistically significant.

The Majorbio Cloud Platform (www.majorbio.com) was used to process the 16S rRNA data. Permutational multivariate analysis of variance (PERMANOVA) was conducted by the R package “vegan” and the permuted *P*-value was obtained from 10,000 permutations. All analyzes were carried out on the level of genus. Alpha diversity (ace and chao) and beta diversity (principal coordinate analysis, PCoA) was performed to explore the similarity or difference of community composition between HSPN and healthy controls. The similarity and overlap of genera in different group were presented in the Veen chart. The relationship between clinical manifestations and each bacterium at genus level (g-bacterium) were assessed by Spearman’s rank correlation or Pearson correlation. Receiver operating characteristic (ROC) with area under curves (AUC) was used to evaluate the potency of classifying HSPN from healthy controls.

## Results

### Participants

In all, 52 participants (26 HPSN, and 26 healthy controls) were recruited in this cross-sectional study. The control group matched the HSPN group in gender (*P* = 1.000) and age (*P* = 0.692). However, indicators closely related to kidney damage are obviously worse in the patient group, including levels of blood pressure, proteinuria, hematuria, serum albumin, creatinine, and eGFR (Table 1). There was no difference in hemoglobin concentration between HSPN and healthy controls (*P* = 0.318).

**Table 1:** Baseline data of HSPN patients and healthy controls

	HSPN (*n* = 26)	Controls (*n* = 26)	P
Male (%)	13 (50.0%)	13 (50.0%)	1.000
Age (years)	25.0 (21.5, 40.5)	31.0 (25.8, 39.7)	0.692
SBP (mmHg)	115.0 (105.5, 125.5)	110.5 (105.8, 116.5)	**0.040**
DBP (mmHg)	79.0 (66.8, 72.0)	70.0 (66.8, 72.0)	**0.004**
u-Pro (g/24 h)	0.90 (0.28, 1.86)	0.07 (0.04, 0.09)	**0.001**
u-RBCs (/Hp)	38.0 (8.0, 149.5)	0.0 (0.0, 1.0)	**0.013**
HGB (g/L)	129.5 (115.5, 136.8)	129.0 (123.0, 133.0)	0.318
Alb (g/L)	40.6 (33.3, 45.2)	44.5 (42.8, 46.5)	**0.016**
sCr (μmol/L)	71.0 (51.0, 108.8)	61.0 (55.0, 70.0)	**0.035**
eGFR (mL/min/1.73m^2^)	109.5 (57.1, 128.1)	118.1 (112.5, 125.8)	**0.011**

Abbreviation: *HSPN* Henoch-Schönlein purpura nephritis, *SBP* systolic blood pressure, *DBP* diastolic blood pressure, *u-Pro* urine protein, *u-RBCs* urinary red blood cells, *HGB* hemoglobin, *Alb* serum albumin, *sCr* serum creatinine, *eGFR* estimate glomerular filtration rate

### Gut microbiota structure of adults with HSPN and healthy controls

**Fig. 1:**
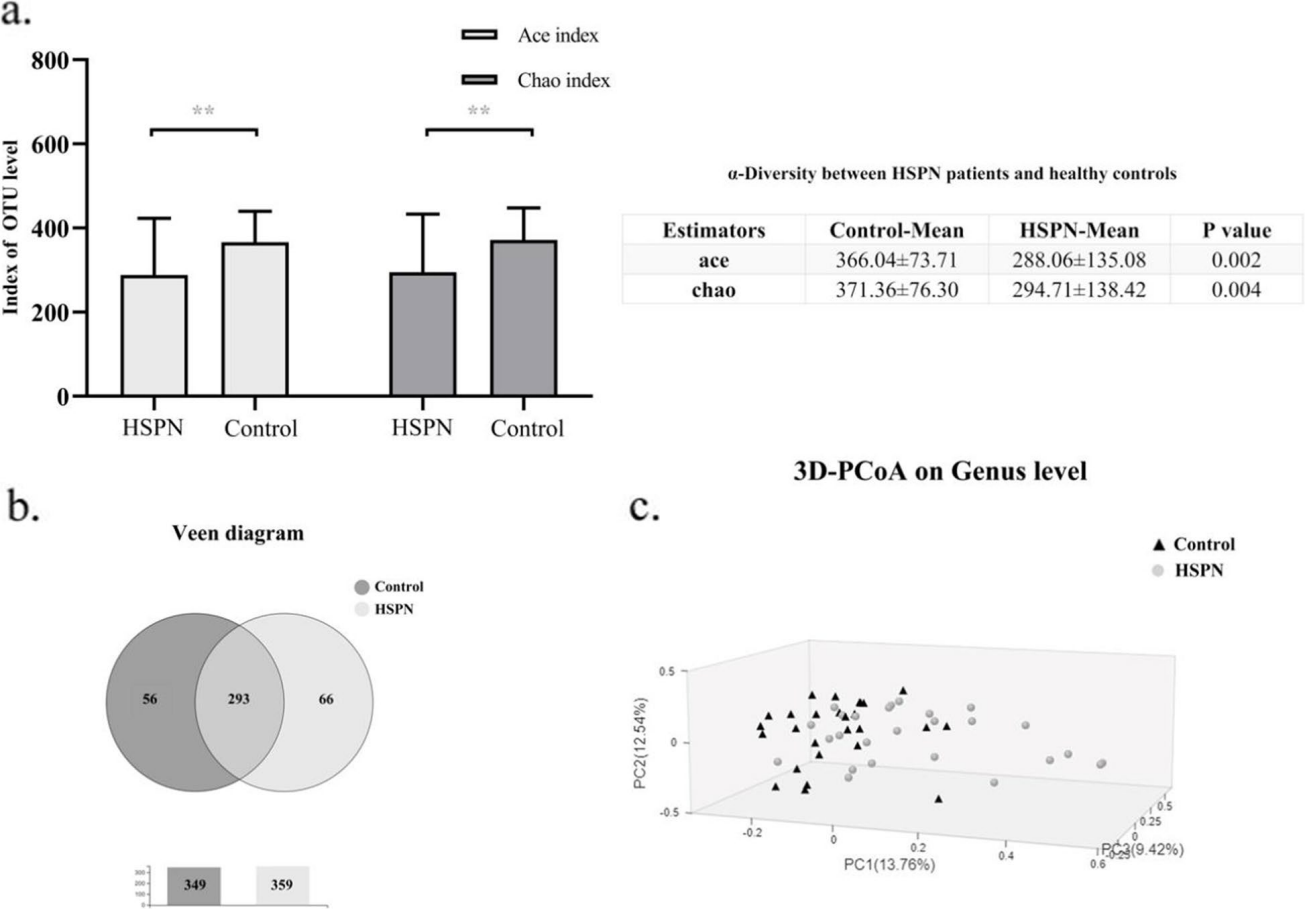
Alpha diversity, Venn diagram and Principle Coordinate Analysis (PCoA) of gut microbiota from HSPN patients and healthy controls at the genus level. (**a**) Alpha diversity between HSPN patients and healthy volunteers. (**b**) Veen diagram. There were 293 genera shared between the two groups, while 66 and 56 genera were respectively specific to the HSPN patients and healthy volunteers. (**c**) The circles and triangles in the PCoA analysis represent the individual samples. The distance between the samples in the figure indicates the degree of difference in the community, showing that the composition of the intestinal bacteria in HSPN patients was substantially different from that of healthy controls

The diversity of the bacterial community was evaluated by alpha diversity (Fig. 1a). The ace index (288.06 ± 135.08) and chao index (294.71 ± 138.42) for patients with HSPN was significantly lower than those in healthy controls (366.04 ± 73.71, 371.36 ± 76.30, respectively), suggesting patients with HSPN had the lower diversity of gut microbiota.

The Veen chart for counting the number of common and unique species in different groups was used to intuitively show the similarity and overlap of genera (Fig. 1b). The results demonstrated that 293 kinds of common genera were identified, but 18.4% (66/359) and 16.0% (56/349) of the unique genera was observed in HSPN and healthy controls, respectively.

The difference of species abundance between groups was reflected by PCoA. It should be noted that in the PCoA diagram, the closer the two sample points are, the more similar the species composition of the two samples, and vice versa. Therefore, Fig. 1c reveals that the overall structure of the gut microbiota is remarkably different between HSPN and healthy controls.

### Differential taxonomic abundance

**Fig. 2:**
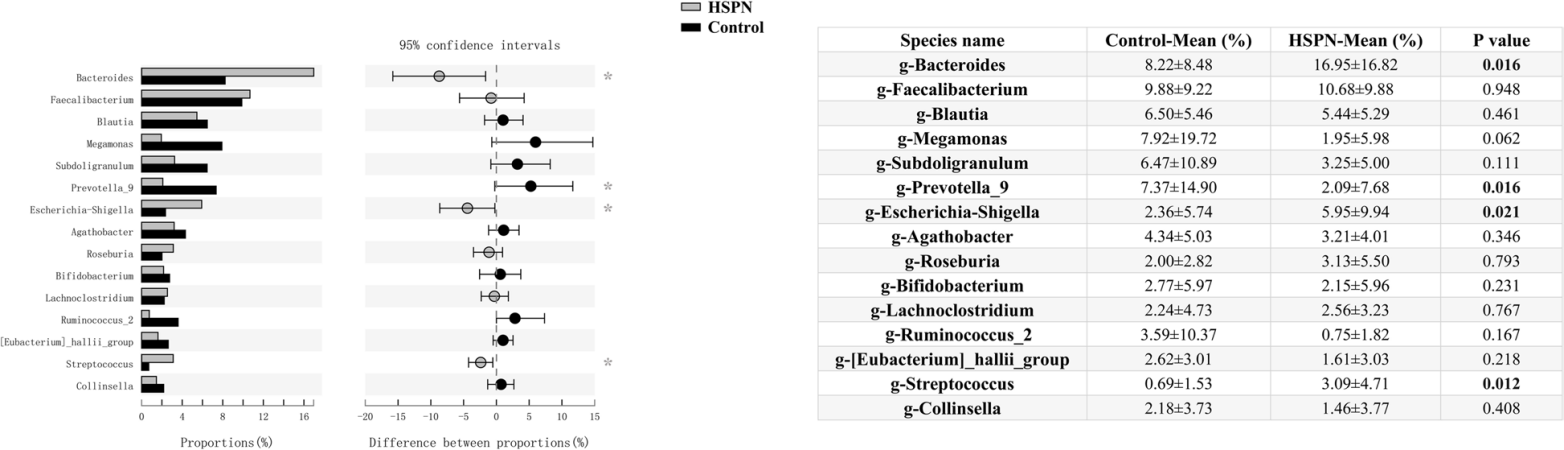
Relative abundance of the gut microbiota between HSPN and healthy control at the genus level

A genus-level comparison between HSPN and healthy controls indicated a significant increase in the proportions of g-Bacteroides (*P* = 0.016), g-Escherichia–Shigella (*P* = 0.021) and g-Streptococcus (*P* = 0.012), and a marked reduction of g-Prevotella_9 (*P* = 0.016) in patients with HSPN (Fig. 2), suggesting that the relative abundance of the predominant bacteria was obviously different between groups.

**Fig. 3:**
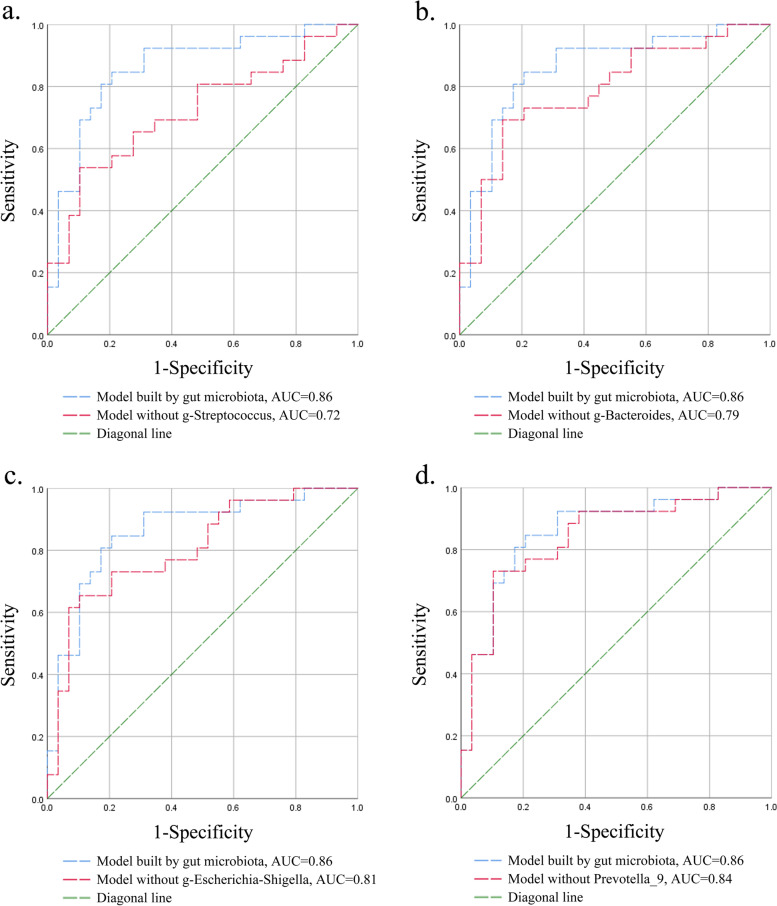
The potency of classifying HSPN from healthy controls, using Receiver operating characteristic (ROC) with area under curves (AUC). The model analyzed by ROC was built by the differentiated gut microbiota. (**a**) Model without g-Streptococcus. (**b**) Model without g-Bacteroides. (**c**) Model without g-Escherichia-Shigella. (**d**) Model without g-Prevotella_9

The differential taxonomic abundance might make sense for distinguishing HSPN from healthy controls. Hence, the ROC curves were developed to illustrate the microbial signature of HSPN (Fig. 3). The AUC of the model built by the differential strains of gut microbiota (g-Bacteroides, g-Escherichia–Shigella, g-Streptococcus and g-Prevotella_9) was 0.86, with an acceptable diagnostic efficacy. The diagnostic performance of each strain was also evaluated by removing it from the model. When one of the strains was removed, the AUC value dropped to 0.72 (without g-Streptococcus), 0.79 (without g-Bacteroides), 0.81 (without g-Escherichia–Shigella), and 0.84 (without g-Prevotella_9), respectively, which implied that the differential taxonomic abundance had the ability to classify HSPN from healthy controls. However, g-Prevotella_9 did not seem to be very valuable for diagnosis of HSPN.

### Connections between gut microbiota and clinical indices

To investigate the relationship between the differential taxonomy at genus level and clinical indices, the Spearman’s rank correlation coefficient was calculated in patients with HSPN. Generally, strong links (Supplementary Table 1) were detected among 4 genera and 6 clinical parameters. The genus Bacteroides was negatively correlated with urine protein level (*r* = − 0.430, *P* = 0.028). A marked inverse correlation could be observed between Prevotella_9 and diastolic blood pressure (*r* = − 0.483, *P* = 0.023). Notably, Streptococcus spp. was positively correlated with the number of urinary red blood cells (*r* = 0.492, *P* = 0.011) and negatively related to serum albumin level (*r* = − 0.474, *P* = 0.014).

Then the patients with HSPN were divided into two groups according to the median levels of g-Streptococcus. However, no significant difference in the clinical indicators were observed. Therefore, we grouped patients into three groups (Low level group, L group; moderate level group, M group; high level group, H group) by quartile (regarded the middle two quarters as the same group). The clinical characteristics are shown in Table 2. It could be found that patients in H group had the remarkably higher levels of proteinuria (2.30 ± 2.43 vs. 0.66 ± 0.55, *P* = 0.050) and urinary blood cells (402.3 ± 508.5 vs. 58.6 ± 128.4, *P* = 0.027), and the significantly lower levels of serum albumin (34.2 ± 13.4 vs. 45.1 ± 6.6, *P* = 0.027), compared with the patients in L group. Simultaneously, the number of urinary blood cells in H group were also higher than that in M group (402.3 ± 508.5 vs. 84.9 ± 120.4, *P* = 0.022). Furthermore, patients were categorized into whether or not hematuria was present (Table 3). It could be easily found that the patients with presentation of hematuria had a significantly higher proportion of Streptococcus (3.6 ± 5.2 vs. 0.8 ± 1.8, *P* = 0.050). Accordingly, it was reasonable to drew a conclusion that Streptococcus spp. might be positively associated with the severity of HSPN.

**Table 2:** The differences of clinical characteristics in the subgroup divided by the quartile range of g-Streptococcus

Parameter	g-Streptococcus					
	Low level group (L)	moderate level group (M)	high level group (H)	P		
	≤0.12%	> 0.12%, but ≤ 4.78%	> 4.78%	L vs. M	L vs. H	M vs. H
Numbers (%)	7 (26.9)	13 (50)	6 (23.1)			
Male (%)	4 (57.1)	5 (38.5)	4 (66.7)	0.642	1.000	0.350
M1 (%)	7 (100)	11 (84.6)	5 (83.3)	0.521	0.462	1.000
E1 (%)	2 (28.5)	2 (15.4)	2 (33.3)	0.587	1.000	0.557
S1 (%)	1 (14.3)	7 (53.8)	4 (66.7)	0.158	0.103	1.000
T1–2 (%)	0 (0)	1 (7.6)	0 (0)	1.000	–	1.000
C1–2 (%)	1 (14.3)	4 (30.7)	4 (66.7)	0.613	0.103	0.319
Age (years)	33.0 ± 11.8	30.6 ± 10.6	24.0 ± 15.0	0.675	0.190	0.274
SBP (mmHg)	133.2 ± 23.2	115.2 ± 14.3	122.3 ± 24.4	0.407	0.975	0.466
DBP (mmHg)	84.4 ± 15.5	78.2 ± 10.5	68.5 ± 11.8	0.576	0.663	0.986
u-Pro (g/24 h)	0.66 ± 0.55	1.04 ± 1.17	2.30 ± 2.43	0.577	**0.050**	0.092
u-RBCs (/Hp)	58.6 ± 128.4	84.9 ± 120.4	402.3 ± 508.5	0.832	**0.027**	**0.022**
HGB (g/L)	131.3 ± 17.7	129.9 ± 12.4	120.6 ± 27.8	0.866	0.326	0.330
Alb (g/L)	45.1 ± 6.6	39.5 ± 5.8	34.2 ± 13.4	0.165	**0.027**	0.206
sCr (μmol/L)	133.4 ± 122.0	72.2 ± 34.0	102.3 ± 60.4	0.220	0.608	0.666
eGFR (mL/min/1.73m^2^)	78.6 ± 33.2	109.7 ± 30.4	100.5 ± 45.7	0.092	0.506	0.455
IgA (μg/mL)	899.8 ± 609.9	987.3 ± 860.9	379.26 ± 134.3	0.846	0.377	0.237
Gd-IgA1 (ng/mL)	767.4 ± 264.3	887.5 ± 389.9	913.3 ± 239.7	0.577	0.643	0.927
Gd-IgA1/IgA (‰)	1.1 ± 0.6	1.7 ± 1.6	4.8 ± 3.2	0.573	**0.015**	**0.018**

Abbreviation: *M* mesangial proliferation, *E* endocapillary proliferation, *S* segmental glomerulosclerosis, *T* tubular atrophy or interstitial fibrosis, *C* crescents, *SBP* systolic blood pressure, *DBP* diastolic blood pressure, *u-Pro* urine protein, *u-RBCs* urinary red blood cells, *HGB* hemoglobin, *Alb* serum albumin, *sCr* serum creatinine, *eGFR* estimate glomerular filtration rate, *Gd-IgA1* galactose-deficient IgA

**Table 3:** The different percentages of the differential gut bacteria in the subgroup divided by levels of hematuria

Parameters	Hematuria		
	Absent (≤ 5/HP)	Present (> 5/HP)	P
**Numbers (%)**	6 (23.1)	20 (76.9)	
**Male (%)**	3 (50)	10 (50)	1.000
**Age (years)**	32.2 ± 9.0	29 ± 12.8	0.580
**g-Bacteroides (%)**	17.7 ± 15.9	14.7 ± 15.1	0.677
**g-Prevotella_9 (%)**	0.5 ± 0.9	2.7 ± 8.8	0.567
**g-Escherichia-Shigella (%)**	2.7 ± 3.5	6.9 ± 11.3	0.382
**g-Streptococcus (%)**	0.8 ± 1.8	3.6 ± 5.2	**0.050**

Our study found that the relative abundance of g-Streptococcus was positively related to the percent of segment sclerosis and crescents, implying that patients with severe pathological lesions tended to have more proportion of g-Streptococcus (Table 4). Although there was no obvious association between g-Streptococcus and IgA or Gd-IgA1, the relationship between g-Streptococcus and Gd-IgA1/IgA could not be neglected (r = 0.790, *P* < 0.001, Table 5). Hence, it was reasonable to drew a conclusion that g-Streptococcus might be related to abnormal expression of IgA in patients with HSPN.

**Table 4:** Correlation analysis of pathological lesions and g- Streptococcus

	M		E		S		S percent		T		C		C percent	
	r	P	r	P	r	P	r	P	r	P	r	P	r	P
g-Streptococcus	0	0.999	0.175	0.488	0.189	0.452	**0.576**	**0.012**	−0.167	0.509	**0.727**	**0.001**	**0.668**	**0.002**

Abbreviations: *M* mesangial proliferation, *E* endocapillary proliferation, *S* segmental glomerulosclerosis, *T* tubular atrophy or interstitial fibrosis, *C* crescents,

**Table 5:** Correlation analysis of serum immunoglobulin A and g- Streptococcus

	IgA		Gd-IgA1		Gd-IgA1/IgA	
	r	P	r	P	r	P
g-Streptococcus	−0.308	0.229	−0.041	0.880	**0.790**	**< 0.001**

Abbreviations: *Gd-IgA1* galactose-deficient IgA1

## Discussion

The microbial populations within fecal samples have been analyzed in our study by using high-throughput sequencing and qPCR analyses. So far, this is the first study to report differences in intestinal flora of adult patients with HSPN. It was indicated that the significantly lower community richness and diversity of fecal microbiota was displayed in HPSN patients. Beta-diversity analysis also supported the fact that the structure of gut microbiota was different between HSPN and healthy controls. Meanwhile, we observed that the microbes were not the same from phylum to genus levels and the Veen chart revealed that the differential bacteria did occur in the patients. All of these implied that there might be intestinal microbiota dysbiosis in adult patients with HSPN. Further investigation found that at the genus level, the relative abundance of Bacteroides, Escherichia–Shigella, and Streptococcus was overrepresented while the proportion of Prevotella_9 was reduced. Generally, Prevotella spp. is thought be a beneficial bacterium, with ability to produce short-chain fatty acids, which is shown to have anti-inflammatory properties in kidney diseases [11, 23]. Conversely, Bacteroides, Escherichia–Shigella, and Streptococcus are considered as the conditioned pathogens that can cause local infection and activate immune and inflammatory responses [17, 24–26]. Accordingly, the major feature of intestinal microbial dysbiosis in patients with HSPN was the decrease of beneficial bacteria and the overabundance of potential pathogenic bacteria, compared with healthy controls.

The differential bacteria observed in HSPN patients were closely linked to clinical manifestations. The most distinctive characteristic was that the relative abundance of Streptococcus spp. was positively associated with active degree of HSPN, confirmed by different statistical methods. It was found that HSPN patients with higher level of Streptococcus spp. were more likely to suffering from hematuria and hypoalbuminemia that were typical clinic signs of HSPN and that might serve as poor predictors [27, 28]. Previous studies have proved that mucosal infections caused by Streptococcus spp. may be followed by an immune-mediated disorder, including HSP, acute glomerulonephritic syndrome, or other extrarenal immune-mediated disorders [29]. It should be noted that most of current researches only focus on throat or skin infections, but few studies have reported whether gastrointestinal tract is also infected by Streptococcus spp. in these patients [30]. Our study firstly proposed that the gastrointestinal tracts of adult patients with HSPN who had no history of respiratory tract infection, were infected by Streptococcus spp., and postulated that the relative abundance of Streptococcus spp. might be closely correlated with severity of HSPN. Although no evidence has been found that the intestinal streptococcal infection may lead to the development of HSPN, some scholars have acknowledged that intestinal mucosal immunity and dysbacteriosis play a crucial role in the pathogenesis of IgA nephropathy which is very similar to HSPN [5, 29, 31]. Meanwhile, mucosa infected by Streptococcus spp. can aggravate inflammatory damage by aggregating T helper cell in the mouse model of IgA nephropathy [25]. Therefore, further studies to investigate the relationship between HSPN and Streptococcus and to explain the specific mechanism of “poststreptococcal” HSPN are badly required.

In theory, the examination of intestinal bacteria from excrement has high value in the management of HSPN. ROC analysis in our study demonstrated that the differential bacteria was of great significance in identifying HSPN from healthy controls, suggesting it could be a new diagnostic method. Previous studies have proposed that detecting the relative abundance of gut microbiota potentially contributes to the pathophysiological diagnosis of kidney diseases, which is very similar to our results [11]. Moreover, our study also noted that the bacterial dysbiosis of HSPN was characterized by the overrepresentation of potential pathogens and reduction of profitable strains. Therefore, the rational use of antibiotics and probiotics may alleviate the patient’s symptoms and improve their prognosis. Several researches have proved that the administration of probiotics or probiotic food is helpful for allergic diseases including HSP, atopic dermatitis and etc. [32, 33]. And patients with chronic kidney diseases can also get benefit from gut microbiota modulation [34]. Besides, the clinicopathological manifestations in the mouse model of IgA nephropathy were significantly improved by using antibiotics with capacity of depleting the gut bacteria [35]. To sum up, we reasonably assumed that the clinical outcomes of adult patients with HSPN might get improved by the combination therapy of current treatments and strategies that reverse the gut microbiota dysbiosis.

Limitations should be noted in this study. Since this is an observational study, we did not take any intervention. So, the view whether HSPN patients could benefit from probiotics or antibiotics cannot be directly confirmed. In addition, as a cross-sectional study, it failed to assess the effect of microflora disturbance on renal prognosis. Of note, it is difficult to conclude that the dysbiosis of gut microbiota affects the progression of HSPN, because gut microbiota of HSPN patients were compared with that of healthy control in present study. Population of gut microbiota will be changed with the progression of CKD with increased levels of serum creatinine and blood urea nitrogen. More importantly, race, environment and diet all affect the formation of gut microbiota. Whether our results were applicable to other ethnics may need further discussion. Therefore, a multiple-center randomized controlled trials with large-scale samples are required to verify our results.

## Conclusion

Our study demonstrated that the dysbiosis of the significantly lower community richness and diversity of fecal microbiota was displayed in HPSN patients, characterized by the overrepresentation of potential pathogens and reduction of profitable strains. Among them, intestinal mucosal streptococcal infection might cause the occurrence of HSPN and be closely related to its severity. A microbial-based management of HSPN might be a potential direction of HSPN in the future.

## Supplementary Information

**Additional file 1.**

## Data Availability

Due to privacy policy, the datasets analyzed in this study are not publicly available but it is available from the corresponding author on reasonable request.
