# Supplementary material for: Intestinal dysbiosis featuring abundance of Streptococcus associates with Henoch-Schönlein purpura nephritis (IgA vasculitis with nephritis) in adult
Source: BMC Nephrol. 2022 Jan 3;23:10. doi: 10.1186/s12882-021-02638-x (PMC8722171; doi:10.1186/s12882-021-02638-x)
Supplement: Supplementary file 1 — Additional file 1. [file 12882_2021_2638_MOESM1_ESM.docx]

|  | g-Bacteroides | | g-Prevotella_9 | | g-Escherichia-Shigella | | g-Streptococcus | |
| --- | --- | --- | --- | --- | --- | --- | --- | --- |
|  | r | P | r | P | r | P | r | P |
| SBP | 0.157 | 0.487 | -0.275 | 0.215 | -0.015 | 0.948 | -0.145 | 0.519 |
| DBP | 0.203 | 0.364 | **-0.483^*^** | **0.023**^*^ | -0.052 | 0.818 | -0.165 | 0.462 |
| u-Pro | **-0.430^*^** | **0.028^*^** | 0.217 | 0.287 | -0.144 | 0.482 | 0.278 | 0.169 |
| u-RBCs | -0.227 | 0.265 | 0.055 | 0.790 | -0.151 | 0.463 | **0.492^*^** | **0.011**^*^ |
| HGB | 0.025 | 0.907 | 0.322 | 0.125 | -0.053 | 0.805 | -0.169 | 0.429 |
| ALB | 0.212 | 0.298 | -0.102 | 0.620 | 0.164 | 0.424 | **-0.474^*^** | **0.014**^*^ |
| sCr | 0.106 | 0.606 | 0.233 | 0.252 | -0.335 | 0.095 | -0.233 | 0.253 |
| eGFR | -0.117 | 0.568 | -0.130 | 0.527 | 0.362 | 0.069 | 0.298 | 0.139 |

**Supplementary Table 1. Spearman analysis of clinical parameters and the genus proportion of gut microbiota.**

Abbreviation: SBP systolic blood pressure, DBP diastolic blood pressure, u-Pro urine protein, u-RBCs urinary red blood cells, HGB hemoglobin, Alb serum albumin, sCr serum creatinine, eGFR estimate glomerular filtration rate.
